# Seroprevalence of Mumps in The Netherlands: Dynamics over a Decade with High Vaccination Coverage and Recent Outbreaks

**DOI:** 10.1371/journal.pone.0058234

**Published:** 2013-03-08

**Authors:** Gaby Smits, Liesbeth Mollema, Susan Hahné, Hester de Melker, Irina Tcherniaeva, Sandra Waaijenborg, Rob van Binnendijk, Fiona van der Klis, Guy Berbers

**Affiliations:** 1 Laboratory for Infectious Diseases and Screening, National Institute of Public Health and the Environment, Bilthoven, The Netherlands; 2 Epidemiology and Surveillance Unit, National Institute for Public Health and the Environment, Bilthoven, The Netherlands; The Australian National University, Australia

## Abstract

Here we present mumps virus specific antibody levels in a large cross-sectional population-based serosurveillance study performed in the Netherlands in 2006/2007 (n = 7900). Results were compared with a similar study (1995/1996) and discussed in the light of recent outbreaks. Mumps antibodies were tested using a fluorescent bead-based multiplex immunoassay. Overall seroprevalence was 90.9% with higher levels in the naturally infected cohorts compared with vaccinated cohorts. Mumps virus vaccinations at 14 months and 9 years resulted in an increased seroprevalence and antibody concentration. The second vaccination seemed to be important in acquiring stable mumps antibody levels in the long term. In conclusion, the Dutch population is well protected against mumps virus infection. However, we identified specific age- and population groups at increased risk of mumps infection. Indeed, in 2007/2008 an outbreak has occurred in the low vaccination coverage groups emphasizing the predictive value of serosurveillance studies.

## Introduction

Mumps was a common worldwide childhood disease before the introduction of routine vaccination in several countries. In general it causes a relatively benign infection, but it can result in considerable morbidity including orchitis, deafness, meningitis and very rarely death [1].

In the Netherlands, a combination vaccine of measles, mumps and rubella virus (MMR) has been part of the national immunization program (NIP) since 1987. The MMR vaccine used in the NIP (BMR-Vaccin®) was produced by the national institute for public health and the environment (RIVM) and later by the vaccine institute of the Netherlands (NVI) in license of MSD. Since 2008 it was replaced by Priorix® from GlaxoSmithKline. In the Netherlands, the MMR vaccine always contained the Jeryl Lynn strain, and is routinely administered at the age of 14 months with a second dose at the age of 9 years. Persons born between 1983 and 1985 were offered the first dose at 4 years of age in a catch up campaign which accompanied the vaccine implementation [2]. A high, since a long period stable, overall MMR vaccination coverage of 96% and 93% for respectively the first and second dose has been reported for 2011 [3]. In spite of this high coverage, several mumps outbreaks have occurred in the Netherlands in the past ten years. The first outbreak was observed in 2004 among a highly vaccinated student population at an international hotel school of which 97% had received at least 1 vaccination [4]. A second in 2007/2008 in low vaccination coverage (LVC) communities where people refuse vaccination for religious reasons (vaccination coverage 80–95%) [3], [5], and a third from 2009 onwards amongst a student population with a vaccination coverage of 81% for at least one dose [6], [7]. Similar outbreaks among vaccinated adolescents and students were documented in several other countries over the last years [8], [9].

To obtain insight into the long term protection of the population in the Netherlands against vaccine preventable diseases and to assess the effect of any changes in the NIP, two large cross-sectional population-based serosurveillance studies have been performed in 1995/1996 (the so-called Pienter1 study) [10] and in 2006/2007 (Pienter2 study) [11].

Here we describe the mumps specific IgG antibody levels in the Pienter2 study for the nationwide sample (NS) and the low immunization coverage sample (LVC). Seroprevalence data are compared with those obtained little over a decade earlier in the Pienter1 study and are discussed in light of the recent outbreaks. Specific attention was paid to age-specific immunity to understand possible causes of mumps vaccine failure.

## Materials and Methods

### Study Population

Two independent, by local ethics committee approved, cross-sectional population-based serosurveillance studies were carried out in the Netherlands between October 1995–December 1996 and February 2006–June 2007 (ISRCTN 20164309). Both studies had a similar design which has been described previously [10], [11]. Similar to the first study, a nationwide sample was drawn from eight municipalities proportional to the number of inhabitants in five geographical regions of approximately equal population size in the Netherlands. Within each municipality an age-stratified sample (0, 1–4, 5–9,…, 75–79 years) of males and females was drawn. The first two age strata were over-sampled due to an expected lower response rate in these groups.

In addition to the nationwide sample, eight municipalities in regions with low vaccination coverage were sampled. These municipalities harbor a relatively high concentration of orthodox reformed individuals who decline vaccination for religious reasons. In the Pienter2 study, 6383 samples were available from the nationwide sample and 1517 samples from the LVC sample.

Participants signed an informed consent form prior to participation and were asked to donate a blood sample at a clinic, to fill in a questionnaire at home and to bring their vaccination certificates. Demographic data were available from all invited individuals.

### Laboratory Methods

Serum samples were stored at −80°C until use. From each sample, 5 µl of serum was used to determine the IgG antibody concentration using the mumps vaccine strain (Jeryl Lynn) as the antigen. The fluorescent bead-based multiplex immunoassay (MIA) using Luminex technology was performed as described before [12]. Briefly, serum samples were diluted 1/200 and 1/4,000 in phosphate buffered saline containing 0.1% Tween 20 and 3% bovine serum albumin. An in-house standard, controls and blanks were included on each plate. Antibody concentrations were obtained by interpolation of the mean fluorescent intensity (MFI) in the reference serum curve and, since no international standard is available, expressed in RIVM units per ml (RU/ml). An antibody concentration of ≥45 RU/ml was used as a criterion for seroprevalence, as previously described [13]. To be able to compare the Pienter1 (measured with ELISA [14]) and Pienter2 study (measured with the MIA [12]) properly, antibody concentrations below 3 RU/ml were set at 1.5 RU/ml for both assays, in spite of the large difference in the lower limit of quantitation between the MIA (0.16 RU/ml) and ELISA (3 RU/ml).

### Data Analysis

Data analysis was performed in SAS 9.1.3 (SAS Institute Inc., Cary, NC, USA). Seroprevalence and geometric mean concentrations in the nationwide sample were estimated by weighting for age, gender, ethnicity and degree of urbanization (table 1) to match the population distribution in the Dutch population at 1^st^ of January 1997 and 1^st^ of January 2007 for the first and second Pienter study, respectively. We adjusted for the two-stage cluster sampling by taking into account the strata (regions) and clusters (municipalities). In the analyses of the LVC sample only the cluster sampling was taken into account and weighting was only done by age and gender. The LVC sample was divided into two groups; one containing the orthodox reformed individuals (i.e., the Reformed Congregations in the Netherlands, the Old Reformed Congregations and the Restored Reformed Church) and the other containing the non-orthodox reformed individuals (i.e., Reformed Bond, Christian Reformed Churches, other Protestant Christians, other or no religion, no information on religion).

**Table 1:** Differences in age and sex specific mumps seroprevalence with 95% confidence intervals (CI) between the Pienter1 and Pienter2 study.

	Seroprevalence Pienter1							Seroprevalence Pienter2						
Age	n	Total (%)	95% CI	Males (%)	95% CI	Females (%)	95% CI	n	Total (%)	95% CI	Males (%)	95% CI	Females (%)	95% CI
0–1 months	51	73	(54–92)	52	(27–77)	83	(66–99)	16	79	(47–100)	55	(3–100)	95	(83–100)
2–3 months	84	39	(25–53)	41	(21–60)	38	(20–55)	63	53	(37–69)	44	(24–65)	62	(37–86)
**4–5 months**	113	**30**	(17–42)	**34**	(12–55)	**25**	(15–36)	53	**7**	(0–13)	**6**	(0–15)	**7**	(0–18)
**6–9 months**	251	**6**	(2–10)	**4**	(0–8)	**10**	(2–18)	137	**1**	(0–3)	**0**		**3**	(0–6)
10–13 months	192	8	(3–13)	4	(0–9)	12	(4–21)	96	6	(0–13)	5	(0–14)	8	(0–19)
14–16 months	57	37	(24–49)	49	(32–65)	23	(7–39)	30	62	(46–78)	61	(40–83)	63	(40–86)
17–23 months	117	92	(88–97)	91	(84–98)	93	(87–100)	58	87	(76–97)	88	(72–100)	86	(72–99)
2	189	89	(85–94)	91	(85–96)	88	(81–95)	117	90	(85–95)	86	(77–95)	95	(90–100)
3	226	93	(90–96)	91	(86–97)	95	(91–99)	143	84	(78–90)	79	(69–90)	88	(81–95)
4	156	91	(87–96)	95	(91–100)	86	(78–95)	146	82	(76–88)	78	(69–87)	86	(77–95)
5	106	88	(82–94)	89	(81–97)	88	(78–97)	95	83	(77–89)	86	(76–96)	78	(67–89)
**6**	122	**92**	(87–97)	**92**	(85–99)	92	(84–99)	107	**76**	(67–85)	**72**	(58–86)	**81**	(70–92)
**7**	102	**89**	(83–95)	**90**	(81–99)	88	(80–96)	129	**72**	(64–79)	**61**	(49–72)	**85**	(75–95)
**8**	130	**94**	(90–98)	**93**	(86–100)	96	(91–100)	129	**71**	(62–81)	**68**	(55–80)	**75**	(63–88)
9	97	98	(96–100)	98	(95–100)	98	(95–100)	160	93	(88–98)	93	(85–100)	92	(84–100)
10	114	97	(95–100)	97	(93–100)	97	(94–100)	85	96	(92–100)	94	(85–100)	98	(94–100)
11	111	96	(93–100)	96	(92–100)	96	(90–100)	96	95	(90–99)	93	(86–100)	96	(90–100)
12	125	93	(86–100)	86	(68–100)	98	(95–100)	92	90	(84–96)	87	(77–97)	94	(87–100)
13	126	95	(91–99)	93	(86–100)	97	(94–100)	78	91	(85–96)	91	(83–99)	89	(77–100)
14	102	97	(94–100)	97	(93–100)	98	(93–100)	81	94	(89–99)	94	(87–100)	94	(87–100)
**15**	108	**94**	(90–98)	**95**	(89–100)	94	(88–100)	65	**87**	(78–95)	**86**	(75–97)	**87**	(74–100)
**16–18**	278	**96**	(94–99)	**94**	(90–98)	99	(97–100)	186	**87**	(83–92)	**85**	(76–93)	**90**	(84–95)
**19–21**	207	**92**	(88–97)	**93**	(87–99)	92	(86–99)	223	**87**	(82–92)	**87**	(78–97)	**87**	(82–92)
22–24	198	95	(92–98)	96	(92–100)	95	(90–99)	188	92	(87–96)	95	(89–100)	88	(82–95)
25–27	232	96	(93–99)	98	(95–100)	94	(90–98)	203	91	(86–96)	95	(88–100)	87	(82–93)
28–30	214	93	(90–97)	90	(84–97)	98	(95–100)	202	91	(86–96)	90	(84–96)	91	(85–98)
31–34	363	95	(91–98)	95	(91–99)	95	(91–98)	277	93	(90–96)	95	(90–100)	91	(87–96)
35–39	500	95	(93–97)	95	(92–98)	95	(92–98)	381	93	(90–96)	94	(91–97)	92	(88–96)
40–44	478	97	(95–99)	96	(92–99)	98	(97–100)	309	95	(93–98)	93	(89–97)	98	(96–100)
45–49	468	97	(96–99)	97	(95–99)	98	(96–100)	332	97	(94–99)	97	(93–100)	97	(94–99)
50–54	494	97	(95–99)	96	(91–100)	98	(96–100)	365	94	(92–97)	91	(86–96)	98	(96–100)
55–59	501	97	(95–99)	97	(94–99)	97	(94–100)	349	97	(95–99)	96	(92–100)	98	(96–100)
60–64	452	98	(97–100)	98	(96–100)	98	(97–100)	416	97	(95–98)	95	(91–98)	99	(98–100)
65–69	465	97	(96–99)	98	(97–100)	97	(94–99)	383	97	(95–98)	98	(95–100)	96	(94–99)
70–74	411	100	(99–100)	100	(99–100)	100	(99–100)	330	97	(95–99)	97	(95–100)	97	(93–100)
75–79	329	99	(98–100)	99	(97–100)	99	(98–100)	263	98	(96–100)	97	(94–100)	99	(98–100)

* Table footnotes: In boldface the ages (4–5 months, 6–9 months, 6, 7, 8, 15, 16–18, 19–21 yrs) with a significant difference in seroprevalence between the Pienter1 and Pienter2 study.

Linear regression analysis was performed to assess the persistence of mumps antibodies after one and two MMR vaccinations. This analysis was restricted to individuals with the Dutch nationality who had received their MMR vaccination(s) according to the Dutch vaccination scheme. The once vaccinated group included 2–8 year olds and the twice vaccinated group included ages of 8 years and older. The association between the antibody concentration and time since first or second MMR vaccination was modeled. The optimal transformation for the variable time since vaccination was determined based on the Akaike Information Criterion.

Logistic regression analysis was performed to asses which determinants were associated with a higher risk of being mumps seronegative. Determinants with a p value ≤0.10 were included in the multivariable analysis and crude and adjusted odds ratios (ORs) and 95% confidence intervals (CIs) were calculated.

For the mumps herd immunity a combined threshold of 86–92% was used. From two studies describing the mumps herd immunity the threshold percentages, 86–88% [15] and 88–92% [16], were combined.

## Results

### Seroprevalence in the Nationwide Sample of the Pienter2 Study

**Figure 1:**
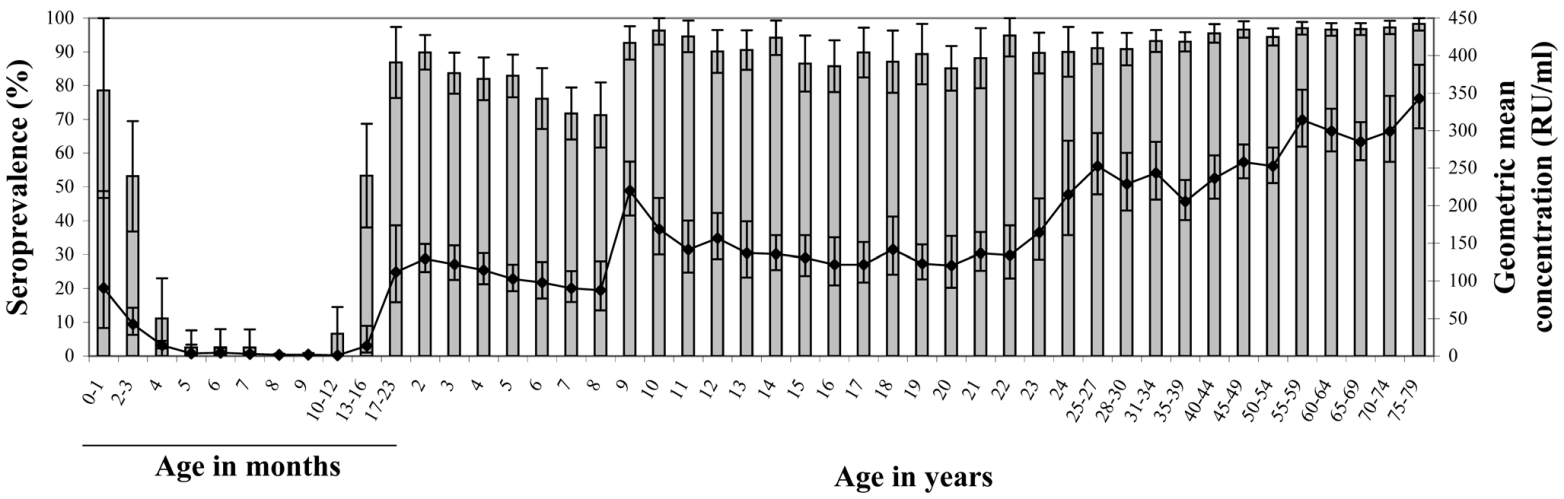
GMC and seroprevalence of the national sample of the Pienter2 study. Age specific geometric mean concentration (GMC, indicated by a line) and seroprevalence (bars) of mumps antibodies of the national sample (NS) from the Pienter2 study. The error bars represent the 95% confidence intervals (CI).

The overall seroprevalence in the nationwide sample was 91% (95% CI 90–92). Seroprevalence of maternal mumps antibodies declined rapidly to 2.5% at the age of 5 months (95% CI 0–8) (figure 1). After the first vaccination at 14 months there was a rapid increase of seroprevalence in the subsequent age groups to 90% (95% CI 85–95) at the age of 2 years. Thereafter it declined gradually to 71% (95% CI 62–81) at the age of 8 years. The administration of the second MMR vaccination at the age of 9 years induced an increase in seroprevalence to 96% (95% CI 92–100) at 10 years of age. Seroprevalence in the twice-vaccinated cohorts decreased gradually to 87% (95% CI 82–92) in the age group of 19–21 years. Participants born before introduction of MMR-vaccination (1987) who were once or twice vaccinated (aged 22–27 years) had a seroprevalence of 91%. Among non-vaccinated individuals, the seroprevalence gradually increased up to 98% in the oldest age groups.

### GMCs in the Nationwide Sample of the Pienter2 Study

The overall GMC in the nationwide sample was 198 RU/ml (95% CI 190–206). The GMC was lower among males than females (183 RU/ml (95% CI 172–194) and 214 RU/ml (95% CI 204–224), respectively, p<0.001). In the first 6 months of life, the GMC of maternal antibodies declined rapidly from 91 RU/ml (95% CI 37–219) just after birth to 5 RU/ml (95% CI 2–11) (figure 1). After the first MMR vaccination at 14 months of age, the GMC increased to 129 RU/ml (95% CI 112–149) at 2 years of age and then declined gradually to 90 RU/ml (95% CI 72–113) by the age of 7 years. The second MMR vaccination around the age of 9 years boosted the GMC to 220 RU/ml (95% CI 187–259), after which the GMC decreased again to 142 RU/ml (95% CI 111–180) by the age of 11 years. Thereafter GMC remained constant between 130–140 RU/ml up to the age of 20 years. Among those aged 22 to 27 years, representing birth cohorts who were not (fully) vaccinated, the GMC showed a sharp increase to about 250 RU/ml. In the older non-vaccinated cohorts the GMC remained constant up to the age of 50–54 years. Thereafter the GMC gradually increased up to almost 350 RU/ml in the oldest age group.

### Comparison of the Pienter1 and 2 Studies

**Figure 2:**
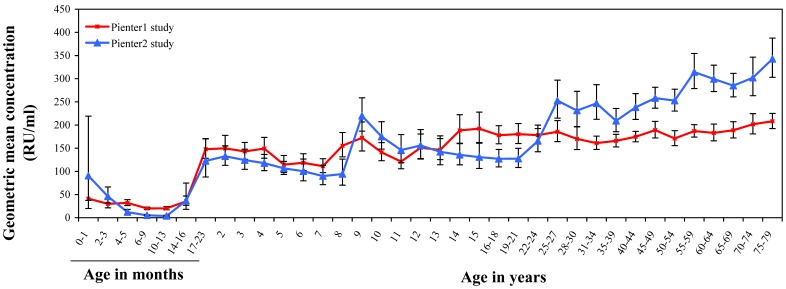
GMC of the national sample of the Pienter1 and Pienter2 study. Age specific geometric mean mumps antibody concentrations (GMCs) of the national sample (NS) of the Pienter 1 (red line) and Pienter 2 (blue line) study. Error bars represent 95% CI’s.

The maternal mumps antibody levels decreased faster in the Pienter2 study than in the Pienter1 study (figure 2). For the once vaccinated cohorts (2–8 years) antibody levels increased in parallel upon the first MMR vaccination, but the levels after vaccination were slightly lower in the Pienter2 study. The second MMR vaccination induced a further increase of antibody levels, which was most striking for the Pienter2 study. As expected, the rise in GMC to a level around 250 RU/ml for the non-vaccinated individuals in the Pienter2 study showed a 10-year shift in concordance with the time period between the two studies. This rise in GMC highlights the difference between the vaccinated and naturally infected cohorts starting in the Pienter1 study from the age of 10–11 years and in the Pienter2 study from 20–21 years. In the unvaccinated naturally infected cohorts above 30 years of age, GMCs were higher in the Pienter2 study compared with the Pienter1 study.

The corresponding seroprevalence in the different age cohorts of the Pienter1 and Pienter2 study (table 1) indicate that the results of both studies were highly comparable, with a few exceptions. A significant lower seroprevalence of maternal antibodies was observed in the Pienter2 study among the 4–5 months (p  = 0.0006) and 6–9 months (p  = 0.012) age groups (table 1). Furthermore, we noticed a significantly lower seroprevalence among participants aged 6, 7 and 8 years in the Pienter2 study as compared to the Pienter1 study (p  = 0.0016, 0.0006 and <0.0001, respectively). Moreover in the 7-years age group from the Pienter2 study, the seroprevalence for boys (61% (95% CI 49–72)) appeared to be significantly (p  = 0.003) lower than for girls (85% (95% CI 75–95)) (table 1). In contrast, no such gender-specific difference for these age groups was observed in the Pienter1 study. Also, a lower seroprevalence was observed in the Pienter2 study for the 15–21 year old age group, which represented antibody levels acquired through vaccination instead of natural mumps infection. Seroprevalence in this age group was 87% in the Pienter2 study, while this was 94% in the Pienter1 study (table 1).

### Waning Immunity in the Pienter2 Study

**Figure 3:**
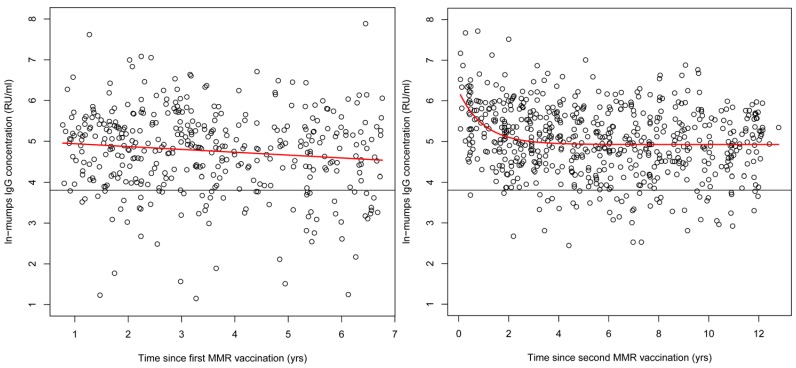
Mumps antibody persistence after vaccination. Persistence of mumps antibodies after MMR-vaccination in the Pienter2 study after the first (left) and second (right) vaccination. In red the fitted model and the black line represents the arbitrary cut-off for seropositivity (45 RU/ml).

After both the first and second MMR vaccination, a significant decline in antibody concentration could be observed in the Pienter2 study (figure 3, p = 0.02 and p<0.0001 respectively). Characteristic for the decline after the first vaccination was the constant decrease in ln-transformed antibody concentration with 0.067 RU/ml per year after vaccination. The initial rise induced by the second vaccination was succeeded by an exponential decrease in ln-transformed antibody concentration in the first 3–4 years. Importantly, in the years thereafter antibody concentrations remained constant around 138 RU/ml.

### Risk Factors in the Nationwide Sample of the Pienter2 Study

Multivariable analysis indicates that younger age groups will have a higher risk at being susceptible for an infection with mumps compared to the oldest, naturally infected age groups because of their lower seroprevalence (table 2). Also, gender, the number of vaccinations and urbanization degree were found to be significant risk factors. A higher risk of being susceptible was observed for males as compared to females (OR  = 1.4 (95% CI 1.1–1.6)), having had no vaccinations as compared to having had two vaccinations (OR  = 4.6 (95% CI 3.0–7.1)) or living in a region with a moderate urbanization degree as compared to living in a region with a high urbanization degree (OR  = 1.5 (1.1–2.1).

**Table 2:** Potential risk factors for mumps seronegativity in the Pienter2 study with corresponding crude and adjusted odds ratios (ORs) and 95% confidence intervals (CIs): results of univariable and multivariable logistic regression analysis.

Potential risk factor	n	% mumps seronegative (95% CI)	Crude OR* (95% CI)	Adjusted OR (95% CI)
**Age**				
0–12 months	359	0.84 (0.80–0.88)	194.1 (125.9–299.2)	199.9 (129.5–308.5)
13–23 months	94	0.27 (0.18–0.37)	13.2 (7.5–23.2)	34.2 (18.1–64.5)
2–8 years	866	0.2 (0.18–0.23)	9.2 (6.4–13.3)	27.3 (16.9–44.2)
9–12 years	433	0.06 (0.04–0.09)	2.5 (1.5–4.2)	9.8 (5.4–17.9)
13–21 years	633	0.12 (0.09–0.14)	4.9 (3.3–7.4)	19.2 (11.2–32.8)
22–30 years	593	0.09 (0.07–0.12)	3.9 (2.5–5.9)	7.0 (4.5–11.0)
31–39 years	658	0.07 (0.05–0.09)	2.8 (1.8–4.4)	2.8 (1.8–4.4)
40–59 years	1355	0.04 (0.03–0.05)	1.6 (1.0–2.4)	1.6 (1.0–2.4)
60–79 years	1392	0.03 (0.02–0.04)	Reference	Reference
**Sex**				
Male	2911	0.15 (0.13–0.16)	1.3 (1.1–1.6)	1.4 (1.1–1.6)
Female	3472	0.11 (0.1–0.12)	Reference	Reference
**Number of vaccinations**				
Zero doses	4135	0.13 (0.12–0.14)	4.5 (3.0–6.9)	4.6 (3.0–7.1)
One dose	1193	0.16 (0.14–0.18)	1.5 (0.97–2.3)	1.5 (0.97–2.3)
Two doses	1055	0.08 (0.06–0.1)	Reference	Reference
**Degree of urbanisation**				
Very high (≥2.500 addresses per km^2^)	1399	0.13 (0.11–0.14)	Reference	Reference
High (1.500–2.500 addresses per km^2^)	2846	0.12 (0.11–0.14)	1.1 (0.9–1.4)	1.2 (0.9–1.5)
Moderately high (1.000–1.500 addresses per km^2^)	803	0.15 (0.13–0.18)	1.4 (1.0–1.9)	1.5 (1.1–2.1)
Low (500–1.000 addresses per km^2^)	589	0.10 (0.08–0.13)	0.8 (0.6–1.2)	0.9 (0.6–1.3)
Very low (<500 addresses per km^2^)	746	0.12 (0.09–0.14)	0.9 (0.6–1.2)	0.9 (0.7–1.3)
**Region**				
North–East	1505	0.13 (0.11–0.15)	Reference	
Central	1121	0.11 (0.09–0.13)	0.9 (0.7–1.2)	
North–West	1527	0.13 (0.12–0.15)	0.99 (0.8–1.3)	
South–West	1125	0.13 (0.11–0.15)	0.9 (0.7–1.2)	
South–East	1105	0.12 (0.10–0.14)	1.0 (0.8–1.4)	
**Ethnicity**				
Dutch	4869	0.12 (0.11–0.13)	Reference	
First generation other Western	153	0.10 (0.06–0.16)	1.8 (1.0–3.2)	
Second generation other Western	292	0.11 (0.08–0.15)	0.8 (0.5–1.3)	
First generation Turkey or Morocco	215	0.14 (0.1–0.19)	1.3(0.8–2.0)	
Second generation Turkey or Morocco	129	0.22 (0.15–0.3)	0.8 (0.5–1.3))	
First generation Surinam. Aruba orNetherlands-Antilles	219	0.1 (0.06–0.14)	1.2 (0.7–1.9)	
Second generation Surinam. Aruba orNetherlands-Antilles	138	0.2 (0.14–0.28)	0.9 (0.5–1.4)	
First generation other non-Western	230	0.12 (0.08–0.17)	1.3 (0.8–2.0)	
Second generation other non-Western	138	0.35 (0.27–0.43)	1.2 (0.7–2.0)	

* Adjusted for age and gender.

### Low Vaccination Sample

**Figure 4:**
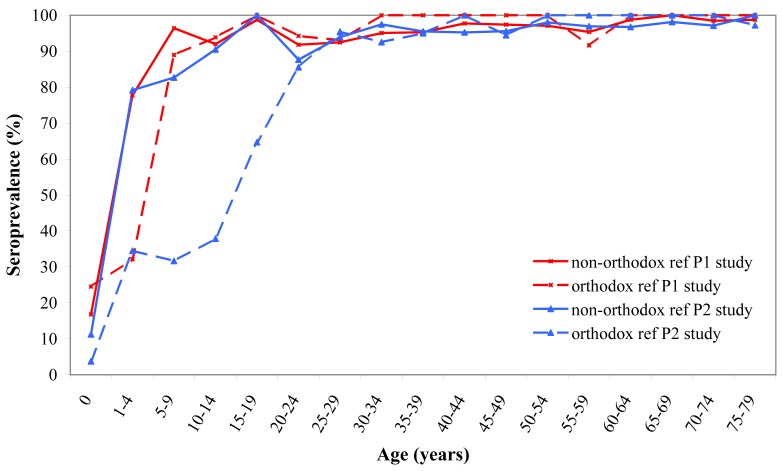
Seroprevalence of the low vaccination coverage sample. Age specific seroprevalence of mumps antibodies of the low vaccination coverage sample (LVC) from the Pienter1 (red line) and Pienter2 (blue line) study.

For the orthodox reformed individuals in the Pienter2 study, the seroprevalence in the younger age cohorts was low, when compared to the same age cohorts of the national sample. By the age of 20–24 years, seroprevalence reached a level of 86% (95% CI 73–98), while a level of 79% (95% CI 73–86) was already reached by the age of 1–4 years in the non-orthodox reformed individuals (figure 4). This difference in seroprevalence reflected the degree of vaccination coverage in the two groups. In the non-orthodox reformed group, vaccination coverage amounted to around 77%, thereby following the trend of the national sample, while in the strictly reformed group, vaccination coverage reached a maximum of 33%.

The seroprevalence of the LVC sample from the Pienter1 study provided a similar pattern, but seroprevalence for the strictly reformed group significantly increased within the age group of 5–9 years. This rise was delayed in the Pienter2 study by approximately 10 years with a rise starting within the age group of 10–14 years (figure 4). For the age cohorts older than 24 years, seroprevalence remained above 90% in as well the Pienter1 as the Pienter2 study for both groups of the LVC.

## Discussion

### National Sample

Here we present the mumps specific antibody levels of a population-based serosurveillance study performed in 2006–2007 (Pienter2 study) in the Netherlands. Overall seroprevalence was high, with two minor gaps in the age cohorts of 5–13 months and 3–8 years. After the first vaccination at 14 months, GMCs remained largely above the defined cut-off value in all age cohorts. In the low vaccination sample, however, the orthodox reformed group reached the seroprevalence level satisfactory for herd immunity (86–92%) [15], [16] only by the age of 20 years and thus much later than the national sample and the non-orthodox reformed groups.

We demonstrated that maternal mumps antibodies have completely disappeared by the age of 6–9 months [17]. Since in the Netherlands the first vaccination is administered 14 months of age, a group of about 100,000 young children is at risk for mumps infection. Nevertheless, no mumps outbreaks have been reported in this group so far, indicating that herd immunity in general is sufficient to prevent transmission of the virus to this group. Probably routine vaccination for almost 20 years, and consequently minimized circulation of the virus together with the herd immunity from the close contacts of these young children (mother, father, young siblings and grandparents) provides protection for this group. Therefore, changing the first MMR vaccination to an earlier age seems not necessary yet but close monitoring of this age cohort is of the utmost importance.

Both vaccinations at 14 months and 9 years clearly induced a sharp rise in mumps antibody concentration in the subsequent age cohorts. However, rapidly after the first vaccination a significant decrease in the antibody levels was observed to even below the herd immunity threshold of 86–92%. This rapid decrease is consistent with results from other studies, where the effectiveness of the mumps vaccine component was generally found to be low [18], [19], [20]. Since the second vaccination is administered not until the age of 9 years, the cohort of 3–8 years old children is left relatively susceptible to mumps virus infection. Whether this implicates that the second vaccination has to be accelerated to an earlier age, remains to be discussed in a broader context and falls outside the scope of this study [17]. The antibody decline in the first years after the second vaccination was faster than after the first vaccination (figure 3), which has also been described in other studies [19], [21], [22]. Thereafter the antibody level remained constant from which we can conclude that the second vaccination appeared to be essential to induce a more sustained humoral response. A similar result was observed by Vandermeulen et al., who showed that two doses of MMRV vaccine provided a significantly better humoral protection against measles and mumps [23]. On the other hand, outbreaks among students (median age 21 years) have recently been observed in the Netherlands [6], [7]. This demonstrates that the presence of antibodies may not confer complete protection after the second vaccination in certain situations (i.e., crowding). This was confirmed by leBaron et al., who found that 12 years after the second MMR vaccination, antibody levels declined to levels similar to pre second MMR vaccination [24]. However, our results demonstrate that 12 years after the second MMR vaccination geomean antibody levels are comparable with those 2 years after the second vaccination.

Most remarkably, 6–8 year old boys seem more susceptible for mumps virus infection than girls of that age. This gender specific difference was not present in the Pienter1 study, although a significant (p<0.001) difference in mumps antibody GMCs between males and females in the age groups over 10 years was observed, with males having lower GMCs than females [14]. Differences between males and females in humoral response to several vaccine components including measles and rubella have been described earlier [25], [26], [27]. However, lower antibody levels do not necessarily imply that the risk of mumps virus infection is higher. It has been demonstrated that persons seronegative for mumps can still exhibit a significant mumps specific lymphoproliferative response even 20 years after their last vaccination [28]. Whether this cellular immune response alone is a sufficient basis for protection against infection with mumps virus, or if it only contributes to protection in combination with circulating antibodies remains to be investigated [29]. However, this might be the reason that so far no outbreaks have been observed among the above-mentioned susceptible cohorts of 6–13 months old children as well as the 3–8 year olds including the 7-year-old boys. Another explanation might be that the applied cut-off value does not entirely correlate with immune protection, as it has not been empirically validated in outbreak situations, which may lead to an underestimation of protective levels. In addition, herd immunity might contribute to protection when seroprevalence has decreased below the supposedly protective percentage in these age cohorts.

In 2009, a mumps outbreak started in the Netherlands among a highly vaccinated student population aged 18–24 years [6], [7]. In the Pienter2 study, a small drop in seroprevalence was observed for the age cohort of 15–21 years, but seroprevalence decreased only just below the herd immunity threshold of 86–92% (table 1). Although in the general population the seroprevalence exceeded this threshold, it recently was documented that mumps virus infection can spread in certain vaccinated age cohorts [30], [31]. The number of students within the 15–21 year age cohort susceptible for an infection could have been large enough to account for an outbreak. The homogenous contact patterns of these students and crowding at parties might have initiated such outbreaks [7]. Moreover, the mumps virus is transmitted via respiratory droplets for which close contact between persons is necessary [32]. Another complementary explanation for the outbreaks could be that the circulating wild type mumps virus (genotype G) responsible for the recent sustained outbreaks differs from the vaccine genotype (A). Although antibodies induced by the Jeryl Lynn strain were found to effectively neutralize also genotype G strains, the antibody levels in general were lower for this wild type virus strain compared with the vaccine strain [33].

### Comparison of the Pienter2 Study with the Pienter1 Study

In the 0–9 year cohorts of the NS the mumps-specific GMCs were found to be higher in the Pienter1 study than in the Pienter2 study. We cannot exclude with certainty that this is an effect of virus circulation which might have still occurred at the time of the Pienter1 study, and could explain the much earlier increase in seroprevalence in the mostly non-vaccinated LVC groups in the Pienter1 study (5–9 year cohort) compared with the LVC groups in the Pienter2 study (20–24 year cohort). The observed ten-year shift in the rise in GMC levels between the vaccinated cohorts and the naturally infected cohorts of the NS was expected and correlates well with the time of MMR vaccine implementation (1987) and the time of the two studies. Apparently, antibody levels induced by vaccination are significantly lower than those induced by natural infection. This was earlier described for measles and rubella [34], [35], [36] and more recently confirmed in a MMR study in Luxembourg [37]. Antibody levels after the two vaccinations are however well above the cut-off value of 45 RU/ml for our assay as determined by the European Seroepidemiology Network [13]. Whether the higher level of antibodies induced by natural infection implies a better protection remains to be investigated.

Remarkable was the difference in GMC between the naturally infected groups in the Pienter1 and Pienter2 study, for the cohorts of 30 years and older. GMCs in these cohorts were significantly (p<0.005) higher in the Pienter2 study than in the Pienter1 study and kept increasing with age in the Pienter2 study. In these older age cohorts in the Pienter2 study a large number of samples were found with very high levels of mumps antibodies, which proved to be mumps specific as was demonstrated by inhibition experiments (results not shown). The discrepancies in GMC levels between the two studies might be due to differences in laboratory methods used. In contrast with the MIA, the samples in the Pienter1 study were measured with an ELISA using only a 1∶100 serum dilution. This limited the upper level of antibody concentrations that could be measured, probably leading to an underestimate of the GMCs in the Pienter1 study. This would mean that differences between Pienter1 and Pienter2 studies for the cohorts of 30 years and older might in reality be smaller whereas for other age groups (i.e., between 1.5 and 5 years and between 14 and 19 years) they might be larger. The public health relevance of this is uncertain since, apart from infants prior to the first MMR, all age groups in Pienter2 had GMCs well above the assumed cut-off for protection.

### Low Vaccination Coverage Communities

In the LVC sample, particularly the cohort of 0–19 years from the strictly reformed religious groups was susceptible for mumps virus infection, with a seroprevalence far below the 86–92% herd immunity threshold. Indeed, in 2007–2009 a genotype D mumps outbreak has occurred within these communities with low vaccination coverage [5], [38]. The median age in this outbreak was 13–15 years, proving the susceptibility of this cohort of the LVC population. This outbreak was limited to the LVC group, indicating that herd immunity in the national sample against this genotype was still sufficient. However, it is important to maintain a seroprevalence level above the 86–92% herd immunity threshold to prevent spreading of a new mumps virus outbreak to the general population.

### Conclusion

The Dutch population is well protected against mumps, although some groups are at risk in the nationwide sample as well as in the low vaccination coverage groups.

The second vaccination seems crucial for obtaining an antibody level that remains protective for a longer period and thereby keeping the seroprevalence in the nationwide sample above the herd immunity threshold of 86–92%.

The predictive value of a serosurveillance study has been emphasized in our country, because shortly after the performance of the study (2007) outbreaks have indeed occurred among the LVC risk groups (2007/2008) and also among the general population (2009). In the latter case, the outbreaks were localized within the student population probably due to social coherence, intense crowding and the reduced seroprevalence in these age cohorts that fluctuates around the herd immunity threshold.
